# Benznidazole and Posaconazole in Experimental Chagas Disease: Positive Interaction in Concomitant and Sequential Treatments

**DOI:** 10.1371/journal.pntd.0002367

**Published:** 2013-08-15

**Authors:** Lívia de Figueiredo Diniz, Julio A. Urbina, Isabel Mayer de Andrade, Ana Lia Mazzeti, Tassiane Assíria F. Martins, Ivo Santana Caldas, André Talvani, Isabela Ribeiro, Maria Terezinha Bahia

**Affiliations:** 1 Laboratório de Doença de Chagas, Departamento de Ciências Biológicas & Núcleo de Pesquisas em Ciências Biológicas, Universidade Federal de Ouro Preto, Ouro Preto, Minas Gerais, Brazil; 2 Instituto Venezolano de Investigaciones Científicas, Caracas, Venezuela; 3 Drugs for Neglected Diseases Initiative, Geneva, Switzerland; Federal University of São Paulo, Brazil

## Abstract

**Background:**

Current chemotherapy for Chagas disease is unsatisfactory due to its limited efficacy, particularly in the chronic phase, with frequent side effects that can lead to treatment discontinuation. Combined therapy is envisioned as an ideal approach since it may improve treatment efficacy whilst decreasing toxicity and the likelihood of resistance development. We evaluated the efficacy of posaconazole in combination with benznidazole on *Trypanosoma cruzi* infection *in vivo*.

**Methods and Findings:**

Benznidazole and posaconazole were administered individually or in combination in an experimental acute murine infection model. Using a rapid treatment protocol for 7 days, the combined treatments were more efficacious in reducing parasitemia levels than the drugs given alone, with the effects most evident in combinations of sub-optimal doses of the drugs. Subsequently, the curative action of these drug combinations was investigated, using the same infection model and 25, 50, 75 or 100 mg/kg/day (mpk) of benznidazole in combination with 5, 10 or 20 mpk of posaconazole, given alone or concomitantly for 20 days. The effects of the combination treatments on parasitological cures were higher than the sum of such effects when the drugs were administered separately at the same doses, indicating synergistic activity. Finally, sequential therapy experiments were carried out with benznidazole or posaconazole over a short interval (10 days), followed by the second drug administered for the same period of time. It was found that the sequence of benznidazole (100 mpk) followed by posaconazole (20 mpk) provided cure rates comparable to those obtained with the full (20 days) treatments with either drug alone, and no cure was observed for the short treatments with drugs given alone.

**Conclusions:**

Our data demonstrate the importance of investigating the potential beneficial effects of combination treatments with marketed compounds, and showed that combinations of benznidazole with posaconazole have a positive interaction in murine models of Chagas disease.

## Author Summary

In this study, we investigated the efficacy of posaconazole in combination with benznidazole against *Trypanosoma cruzi* acute infections in mice, to support the potential clinical evaluation of such combination therapy for Chagas disease. The curative action of benznidazole/posaconazole combinations was explored in an established acute infection model with the Y strain in which benznidazole and posaconazole treatments induced a 70% and 80% cure rate, respectively, when administered alone at optimal doses. When tested in combination, a 80% to 90% cure rate was detected in mice receiving 25, 50 or 75 mpk of benznidazole, plus 5 or 10 mpk of posaconazole, while treatment with the sub-optimal doses of the drugs given alone induced only 0–43% cures, indicating synergistic effects. Finally, sequential short (10 days) treatments with benznidazole (100 mpk) followed by posaconazole (20 mpk) led to an 80% cure rate, comparable with full-length treatments with either drug given alone, while no cures were observed for short treatments with single drugs. Our results demonstrate that it is possible to achieve the same or better therapeutic effect using lower dosages of posaconazole and benznidazole in combination, decreasing treatment costs and potential toxicity.

## Introduction

The use of a DNA probe targeted against *Trypanosoma cruzi* kinetoplast DNA extracted from mummified Andean humans demonstrated that Chagas disease was highly prevalent in the Southern Andean coastal area 9,000 years ago [1]. Currently, an estimated 8 to 10 million people are infected with *T. cruzi* from Mexico through Central and South America, with 28 million remaining at risk of infection [2]. In addition, international migrations have resulted in a significant number of infected individuals now residing in non-endemic countries [3]. Progress of vector control endemic areas remains uneven [4], and little progress has been made toward the treatment of already infected people [5].

Current specific chemotherapy for Chagas disease, based on nitroheterocyclic compounds, is unsatisfactory. Long-term longitudinal studies using benznidazole or nifurtimox have shown that the earlier diagnosis is made and specific treatment is started, the better are the chances of a complete parasitological cure. In congenital Chagas disease, early treatment of newborns with benznidazole or nifurtimox is effective in 66% to 100% of the cases [6]. In clinical trials performed during the acute phase of the infection, a 40% to 76% rate of parasitological cures has been attained [7], [8], [9]. Long-term studies also showed significant success for chemotherapy with benznidazole among children and adolescents with short-term chronic infections [10], [11], [12]. In different regions of Bolivia, however, the benznidazole-treatment of children and adolescents induced cure rates of only 0% to 5.4%, when assessed through serology over a period of 18–60 months [13].

No consistently effective treatment exists for the established chronic forms of the disease, which is currently the most common clinical presentation in both endemic and non-endemic areas. Some studies showed improved clinical and serological evolution of patients treated with benznidazole, as compared with untreated chronic patients, but with variable and limited results in some settings [9], [14], [15], [16], [17], [18], [19]. Safety and tolerability has been considered an important constraint to treatment in adult patients. Based on these antecedents, the development of new drugs and evaluation of the impact of trypanocidal treatments in preventing morbidity remain key challenges for Chagas disease control and health care [5]. Still, since the introduction of nifurtimox and benznidazole for the chemotherapeutic treatment of Chagas disease few other compounds have been assessed in humans [5].

Ergosterol biosynthesis inhibitors (EBI) are currently among the most promising anti-*T. cruzi* agents. Their mechanism of action is based on the essential *T. cruzi* requirement for endogenous sterols for survival and proliferation and its inability to use the abundant supply of cholesterol available in mammalian hosts [5]. Despite the significant *in vitro* and *in vivo* activity of these novel azole derivatives against *T. cruzi* and the absence of cross-resistance with currently available drugs, the response to drug treatment varies strongly among different parasitic strains [20], [21], [22], [23], [24]. Two of such compounds, posaconazole and E1224 (a pro-dug of ravuconazole), are currently undergoing Phase 2 (proof-of-concept) clinical trials for the specific treatment of chronic Chagas disease (http://clinicaltrials.gov/show/NCT01377480 and http://clinicaltrials.gov/show/NCT01489228).

Combination therapy can be a valuable way to improve treatment efficacy due its capacity to reduce dose levels and toxicity and to prevent the potential development of resistance to anti-infective drugs [25]. Azole derivatives used in combination with EBIs acting at other steps of the sterol biosynthesis pathway were shown to have synergistic anti-*T. cruzi* effects *in vitro* and *in vivo* [26], [27]. These results emphasize the importance of identifying compounds already on the market (such as the recently registered posaconazole as well as benznidazole and nifurtimox) to explore their potential use in combination for the specific treatment of Chagas disease. In this study, we investigated the anti-*T. cruzi* efficacy of combinations of benznidazole and posaconazole, used in concomitant or sequential therapy, in an experimental murine model of acute Chagas disease to support the potential clinical evaluation of such combination therapies.

## Materials and Methods

### *Trypanosoma (Schizotrypanum) cruzi* stocks

*T. cruzi* Y (DTU II) and VL-10 (DTU II) [28] strains, previously characterized by Filardi & Brener [29] as partially resistant (Y) and resistant (VL-10) to benznidazole, were used in the present study. These strains were obtained from the *T. cruzi* collection at Chagas Disease Laboratory, Federal University of Ouro Preto (UFOP).The original isolates [30], [31] have been maintained as trypomastigote forms in liquid nitrogen, and periodically transferred to mice, and refrozen, with full retention of their biological and drug susceptibility characteristics.

### Drugs

The following drugs were commercially purchased from, or provided by their respective pharmaceutical companies: (i) benznidazole – 2-nitro-imidazole-(N-benzil-2-nitro-1-imidazoleacetamide (Benznidazol, LAFEPE – Laboratório Farmacêutico do Estado de Pernambuco); (ii) posaconazole – (2)-4-[4-[4-[4-[[(2Rcis)-5-(2,4-difluorophenyl)-tetrahydro-5-(1H-1,2,4-triazol-1-ylmethyl)furan-3-yl]methoxy]phenyl]-2,4-dihydro2-[(S)-1-ethyl-2(S)-hydroxypropyl]-3H-1,2,4-triazol-3-one (Noxafil, Schering Plough Research Institute) and (iii) cyclophosphamide (*N*,*N*-bis(2-chloroethyl)-1,3,2-oxazaphosphinan-2-amine 2-oxide (Genuxal, Asta Medica Oncologica).

### *In vivo* assays

Female Swiss mice (18–24 g) were obtained from the Animal Facility at the Federal University of Ouro Preto, Minas Gerais, Brazil, and maintained in a temperature-controlled room with access to water and food *ad libitum*. Animals were inoculated with 5.0×10^3^ blood trypomastigotes of the *T. cruzi* Y or VL-10 strains. After four days, tail blood was examined for the presence of parasites. Only when *T. cruzi* was detected microscopically, were the mice submitted to a specific treatment.

### Dose-response experiment

Infected animals were divided into groups of six and received drugs (benznidazole, posaconazole) alone or in different combinations. All compounds were suspended in distilled water using 2% methyl-cellulose (Sigma), and each animal received 0.2 mL of drug suspension daily by gavage for 7 consecutive days. For the treatment with each drug alone, three doses were selected: (i) the dose able to induce a parasitological cure in a longer treatment course, as previously determined by Filardi & Brener [29] and Molina et al. [20]; (ii) half and (iii) one-fourth of that curative dose. The curative dose in infected mice was 100 mg/Kg/day (mpk) for benznidazole and 20 mpk for posaconazole.

The drug combinations consisted of benznidazole plus posaconazole at the following dosages: 25, 50 or 100 mpk of benznidazole in combination with 5, 10 or 20 mpk of posaconazole. Treatment efficacy was assessed based on three parameters: parasite clearance, pre-patent parasitemia interval, and mortality. Parasitemia and mortality were checked every day during and until ten days after treatment and every other day afterwards until 30 days after treatment.

### Curative activity

A set of experiments was designed to determine the capacity of benznidazole and posaconazole, given alone or in combination (concomitant or sequential) treatments to induce parasitological cure in mice infected with the Y strain of *T. cruzi* (partially resistant to benznidazole) [29].

The concomitant drug combinations consisted of benznidazole plus posaconazole at the following doses: 25, 50, 75 or 100 mpk of benznidazole in combination with 5, 10 or 20 mpk of posaconazole. Results were compared to those reached with benznidazole and posaconazole monotherapies and with infected and non-infected control groups. The drugs were administered for 20 consecutive days upon detection of parasitemia, which occurs on the 4^th^ day post-inoculation.

A second set of experiments was designed to determine the efficacy of benznidazole plus posaconazole combination therapy to induce survival and parasitological cure in mice infected with VL-10, a benznidazole-resistant *T. cruzi* strain [29]. Due to the high benznidazole-resistance of VL-10 strain, higher doses of the drugs were used, both alone and in combination. Thus, animals were treated with 100 mpk of benznidazole and/or 20 mpk of posaconazole b.i.d. (Q12; total of 40 mpk per day) for 20 days. The drug combinations consisted of benznidazole plus posaconazole at the following dosages: 50 or 100 mpk of benznidazole in combination with 10 or 20 mpk of posaconazole. Benznidazole was administered as a single daily dose and posaconazole twice daily (5 or 10 mpk b.i.d. Q12). Results were compared to those reached with benznidazole or posaconazole monotherapy treatments and with control groups, in infected and non-infected mice.

The sequential drug therapies consisted of benznidazole (100 mpk) administered for 10 days followed by posaconazole (20 mpk) for another 10 days and the inverse sequence. Results were compared to those reached with benznidazole and posaconazole given alone for 10 and 20 days and with infected and non-infected control groups.

### Assessment of parasitological cure

The parasitological cure was determined following the methodology standardized by Caldas *et al* [32], based on a battery of two independent tests: fresh blood examination before and after cyclophosphamide immunosuppression (CyI), followed by PCR assays performed on blood samples from mice with negative parasitemia at the 1^st^ (before the CyI) and 6^th^ month after treatment. Animals showing negative results in the two tests were considered cured.

Fresh blood examination - parasitemia of the animals was evaluated during and up to the 30^th^ day post-treatment to determine the natural reactivation of infection. Animals that did not present reactivation of parasitemia after treatment were submitted to CyI, which consisted of three cycles of 50 mg of cyclophosphamide/kg of body weight, for four consecutive days, with intervals of three days between each cycle. The parasitemia of these animals was evaluated during the CyI cycles, and for 10 days following immunosuppression. Animals did not present reactivation of parasitemia after CyI were bled 180 days after treatment for PCR assay.

PCR assay. Mice were bled from the orbital venous sinus and 200 µL of blood were collected 30 and 180 days after the treatment ended. PCR was performed only on samples from animals with negative parasitemia in fresh blood examination. DNA extraction and PCR were performed according to Gomes *et al.* [33] with some modifications. The primers used for the parasite minicircle amplification were: S35 5′-AAATAATGTACGGG(T/G)GAGATGCATGA-3′ and S36 5′-GGGTTCGATTGGGGTTGGTGT-3′ [34]. Thirty-five amplification cycles were carried out in a Research Programmable Thermal Controller (MiniCycler). The cycles consisted of an initial denaturation of 5 min at 95°C followed by 35 cycles of 1 min at 95°C for denaturation, 1 min at 65°C for primer annealing and 1 min at 72°C for primer extension. Five microliters of the PCR product were analyzed by electrophoresis on a 6% polyacrylamide gel and visualized by silver staining. Positive and negative blood samples and reagent controls were processed in parallel for each assay, and all experiments were conducted under controlled conditions. To avoid contamination, DNA extraction, mixing, and electrophoresis were performed in separate, delineated areas. To confirm the absence of inhibition factors, an internal control corresponding to a segment of the murine TNF-α gene was amplified [35].

### Ethics

All procedures and experimental protocols were conducted in accordance with the guidelines issued by the Brazilian College of Animal Experimentation (COBEA) and approved by the Ethics Committee in Animal Research at UFOP (number 2009/16 and 2011/76).

### Statistical analysis

To evaluate variations in the levels of parasitemia among animals treated with each drug alone or in combination, the data were converted using a logarithmic transformation and tested using the Analysis of Variance, with the group comparison performed by using Tukey's Multiple Comparison Test. Differences were considered significant when p<0.05.

## Results

The parasitemia levels and mortality rate of animals infected with the *T. cruzi* Y strain were assessed. All untreated animals presented high levels of parasitemia, which peaked on the 8^th^ day post-infection, and mortality occurred on average at 15 days post-infection (Table 1). The effects of half and one-fourth of the optimal dose for each drug (100 mpk of benznidazole or 20 mpk of posaconazole), given daily as monotherapy for 7 days, on the evolution of the infection in mice were then evaluated and compared with those in mice that received the full dose. The results (Table 1) showed that all compounds had a dose-dependent trypanocidal effect, leading to a transient suppression of parasitemia with a subsequent relapse that gave rise to a peak level inversely related to the drug dose. The optimal dose of each compound was able to significantly reduce the peak levels of parasitemia when compared with the sub-optimal doses (p<0.05, see Table 1). Of note, the peak parasitemias (compared with untreated animals) in mice treated with all doses of posaconazole were consistently lower than those in benznidazole-treated animals, confirming the superior efficacy of the former drug. In addition, the survival of treated animals also presented a dose-dependent response, with only mice receiving the optimal dose of the compounds having 100% survival at 30 days post-treatment. However, those animals receiving an intermediary dose of posaconazole (10 mg) also presented 100% of survival (Table 1). We next evaluated the anti-*T. cruzi* activities of the combinations of benznidazole/posaconazole (100, 50 or 25 mpk of benznidazole with 20, 10 or 5 mpk of posaconazole, respectively for 7 days) and compared them with the results obtained with the same doses of each individual drug given alone. At the doses tested, each monotherapy pair produced statistically-indistinguishable parasitemia levels and the combinations being consistently either statistically significant, or near significantly, different from each monotherapy, suggesting a positive drug interaction. This was most evident in combinations of sub-optimal doses of the drugs (benznidazole at 25 mpk with posaconazole 5 mpk and benznidazole at 50 mpk with posaconazole 10 mpk), which reduced the parasitemia levels to values significantly lower than those obtained with the drugs when given alone, even at the optimal doses, and led to 83.3 to 100% survival (Table 1).

**Table 1:** Efficacy of benznidazole (Bz) and posaconazole (Ps) treatments for 7 days (monotherapy or combination) in murine model of acute *Trypanosoma cruzi* infection^1^.

Experimental groups	Parasitemia clearance (doses)	Number of surviving/total number of animals	Maximum level of parasitemia until 30 days after treatment×10^3^ (mean±SD)
Uninfected	-	6/6	-
Untreated	-	0/6	1,424.6±507.1
Bz 100 mg/Kg/day	1.83±0.75	6/6	95.6±46.8
Ps 20 mg/Kg/day	1.5±0.54	6/6	74.8±38.1
Bz+Ps (100+20 mg/Kg/day)	1.0±0	6/6	13.2±3.9*
Bz 50 mg/Kg/day	1.33±0.51	4/6	174.8±192.8
Ps 10 mg/Kg/day	1.17±0.41	6/6	101.2±1,102.5
Bz+Ps (50+10 mg/Kg/day)	1.4±0.55	5/6	20.1±99.6*
Bz 25 mg/Kg/day	ND	2/6	418.6±518.23
Ps 5 mg/Kg/day	1.17±0.41	4/6	240.2±338.2
Bz+Ps (25+5 mg/Kg/Day)	1.16±0.41	6/6	36.6±28.4*

1 Swiss female (n = 6) weight 20 to 24 g were inoculated with 5×10^3^ trypomastigotes (Ystrain).

Treatment was started at 4^th^ day after inoculation followed by 7 daily doses and the drugs were administered orally.

* Significant difference in relation to benznidazole monotherapy treatment at same dose.

ND – Not detected.

**Figure 1:**
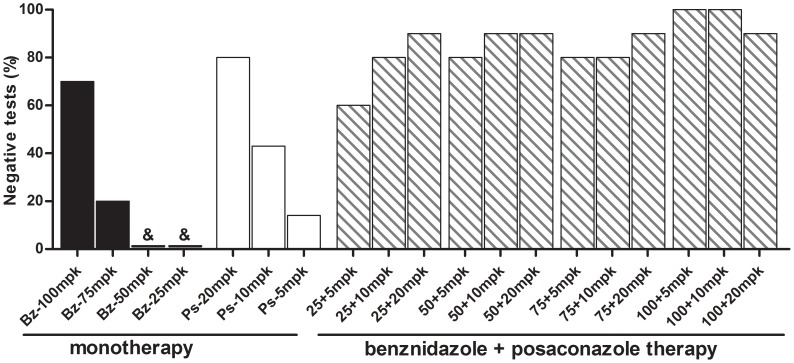
Anti-*Trypanosoma cruzi* activity of benznidazole plus posaconazole combination therapy. Percentage of positive results of fresh blood examination (before and after cyclophosphamide immunosuppression) and PCR assay (performed at 1^st^ and 6^nd^ month post-treatment) from mice infected with *Trypanosoma cruzi* Y strain. Animals were treated with daily doses 25, 50, 75 or 100 mg/kg of bodyweight (mpk) of benznidazole (Bz) and 5, 10 or 20 mpk of posaconazole (Ps) alone or combination for 20 consecutive days. &: all mice had positive results.

The curative activity of benznidazole/posaconazole combinations was explored in an established infection with Y strain in which benznidazole and posaconazole treatments induced 70% and 80% cure rates, respectively, when administered alone at the optimal doses (100 mpk for benznidazole and 20 mpk for posaconazole) for 20 days (Figure 1). As expected, the treatment with sub-optimal dosages of benznidazole and posaconazole led to smaller or nil cure rates. The sub-optimal dosages of benznidazole cured none of the Y strain-infected mice treated with 25 or 50 mpk, while 75 mpk induced just 20% cure rate. Similarly, posaconazole 5 mpk and 10 mpk induced just 14% and 43% cure rates. However, when tested in combination 80% to 90% cure rates were detected in mice receiving 25, 50 or 75 mpk of benznidazole, plus 5 or 10 mpk of posaconazole (Figure 1). Such results clearly indicate synergistic effects in the combined action of the drugs, particularly at the lower doses, as the effects observed with the drug combinations were more than the sum of the effects of the drugs when used alone. Only in animals treated with 25 mpk of benznidazole plus 5 mg of posaconazole was the cure rate (60%) smaller than that observed in animals treated with optimal doses of the drugs alone, but again the cure rate was higher than the sum of those obtained in animals treated with the same doses administrated alone. Interestingly, a cure rate of 90% to 100%was detected in mice treated with an optimal dose of benznidazole (100 mpk) in combination with any of the doses of posaconazole (5, 10 or 20 mpk). The drug combinations were well tolerated by animals and no mortality was detected among animals receiving such treatments. Additionally, no differences in weight gain were found among treated and non-infected animals during the evaluated period (data not shown).

**Figure 2:**
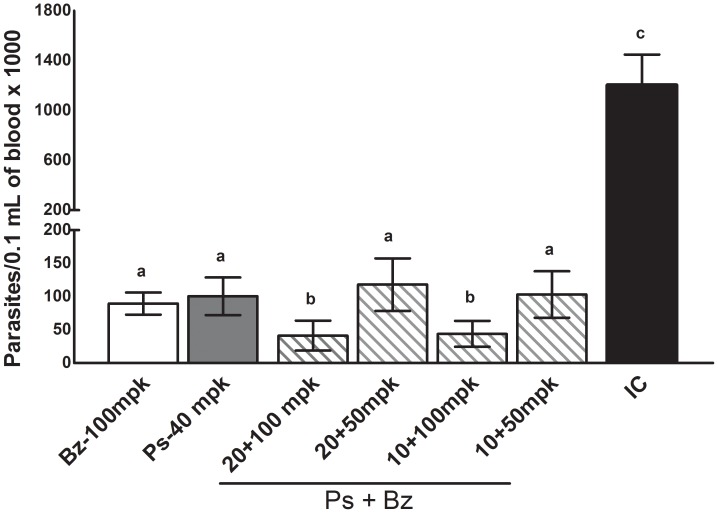
Benznidazole plus posaconazole therapy against VL-10 benznidazole resistant *Trypanosoma cruzi* stock. Maximum number of trypomastigote forms detected in the peripheral blood of mice infected with *Trypanosoma cruzi* VL-10 strain and treated with 100 mg/kg of bodyweight (mpk) of benznidazole (Bz) or 40 mpk of posaconazole (Ps) and with Bz plus Ps combination at the following dosages: 50 or 100 mpk of Bz in combination with 10 or 20 mpk of Ps for 20 consecutive days. Benznidazole was given daily and posaconazole twice a day (b.i.d.). IC – infected and untreated animals. a, b, c - different letters indicate significant differences, and the same letters indicates similar values among parasitemia levels.

Given the high efficacy of benznidazole/posaconazole combinations against the partially benznidazole-resistant Y strain, further experiments were carried out using the highly (100%) benznidazole-resistant strain VL-10 [29]. Due to the high intrinsic resistance of this strain, higher doses of both benznidazole and posaconazole were used in these experiments. Benznidazole or posaconazole were unable to induce parasitological cure in mice infected with the VL-10 strain when administered alone at 100 mpk or 40 mpk (20 mpk b.i.d.), respectively for 20 days (Table 2), since parasitemia reactivation was detected in all mice after the end of treatment. On the other hand, the parasitemia reactivation was detected only in 50% of animals that received 100 mpk of benznidazole in combination with 10 or 20 mpk of posaconazole (Table 2). However, 20–30% (2/10–3/10) of animals that received these combined doses had negative results in both the fresh blood examination and PCR assays, indicating parasitological cures. Consistently, all treatments induced a reduction of parasitemia levels in comparison with infected and untreated animals (p<0.05), but the parasitemia levels detected in peripheral blood of animals receiving combined treatments of benznidazole 100 mpk with 10 or 20 mpk of posaconazole were significantly lower in comparison with those treated with benznidazole or posaconazole alone (*p*<0.001) (Figure 2). The results emphasize that the combination therapy with benznidazole and posaconazole is more effective in reducing circulating parasite levels, increasing survival and inducing parasitological cure than the sum of the effects of the drugs given alone, even against drug-resistant organisms.

**Table 2:** Assessment of cure by benznidazole (Bz) and posaconazole (Ps), administered in monotherapy or concomitant combinations, in a murine model of *Trypanosoma cruzi* infection^1^.

Experimental groups	Number of surviving/total number of animals	Number of negative FBE^2^/number of mice	Number of negative blood PCR^3^ sample/number of mice	Total of negative assays/number of mice
Uninfected	10/10	10/10	10/10	10/10
Untreated	4/10	0/6	0/6	0/6
Bz 100 mg/Kg/day	10/10	0/10	0/10	0/10
Ps 40 mg/Kg/day	10/10	1/10	0/1	0/10
Bz+Ps (100+20 mg/Kg/day)	10/10	5/10	3/5	3/10
Bz+Ps (50+20 mg/Kg/day)	10/10	0/10	0/10	0/10
Bz+Ps (100+10 mg/Kg/day)	10/10	5/10	2/5	2/10
Bz+Ps (50+10 mg/Kg/day)	10/10	0/10	0/10	0/10

1 Swiss female (n = 10) weight 20 to 24 g were inoculated with 5×10^3^ trypomastigotes (VL-10 strain).

Treatment was initiated at 7^th^ day after inoculation followed by 20 days and the drugs were administered orally. Benznidazole was given once daily and posaconazole administered twice a day (b.i.d.).

2 FBE – fresh blood examination performed before and after cyclophosphamide immunosuppression.

3 PCR assay was performed in the 1^st^ and 6^th^ month after treatment.

Based on the encouraging results obtained with the drugs used in concomitant combination, we investigated the effects of the drugs when given in sequential treatments in the same experimental model. Interestingly, the efficacy of the treatments was related to the order of drug administration: it was found that treatment with benznidazole at 100 mpk for 10 days followed by posaconazole at 20 mpk for another 10 days induced 80% of parasitological cure, indistinguishable from the effects of the drugs given alone at the same doses over the full 20-days treatment, but when the order of treatment was reversed (posaconazole at 20 mpk for 10 days followed by benznidazole at 100 mpk for another 10 days) the percent of cures dropped to 30% (Table 3). On the other hand, no cures were observed when animals were given benznidazole 100 mpk or posaconazole 20 mpk for 10 days.

**Table 3:** Assessment of cure by benznidazole (Bz) and posaconazole (Ps), administered in monotherapy or sequential combinations, in a murine model of *Trypanosoma cruzi* infection^1^.

Experimental groups	Number of surviving/total number of animals	Number of negative FBE^2^/number of mice	Number of negative blood PCR^3^ sample/number of mice	Total of negative assays/number of mice
Uninfected	10/10	10/10	10/10	10/10
Untreated	0/10	0/10	ND^4^	0/10
Bz 100 mpk^4^/10 days	7/7	0/7	ND^5^	0/7
Bz 100 mpk/20 days	10/10	8/10	7/10	7/10
Ps 20 mpk/10 days	6/7	0/7	ND^6^	0/7
Ps 20 mpk/20 days	10/10	10/10	8/10	8/10
Bz 100 mpk followed by Ps 20 mpk	10/10	10/10	8/10	8/10
Ps 20 mpk followed by Bz 100 mpk	10/10	3/10	3/10	3/10

1 Swiss female (n = 10) weight 20 to 24 g were inoculated with 5×10^3^ trypomastigotes (Y strain).

Treatment was initiated at 4^th^ day after inoculation followed by 10 or 20 daily doses and it was being orally administered.

2 FBE – fresh blood examination performed before and after cyclophosphamide immunossupression.

3 PCR assay was performed in the 1^st^ and 6^th^ month after treatment.

4 mpk – milligrams/kilogram/day.

5 All mice died before 30 days of infection.

6 All mice had positive results in fresh blood examination.

## Discussion

Although encouraging advances have been made in the control of vectorial and transfusional transmission of *T. cruzi*, there is still a need to develop safe, efficient and affordable new specific treatments for Chagas disease, particularly in its chronic stage [5], [36]. Few drugs are available for the treatment of this neglected disease and they have inadequate safety and tolerability profiles. Research and development of new specific medicines has been neglected for too many years, despite substantive advances in our understanding physiology and biochemistry of the etiological agent and the mechanisms of pathogenesis.

Recent studies have shown that compounds such as the sterol C14α demethylase inhibitor posaconazole provide a high percentage of parasitological cures in several animal models of acute and chronic Chagas disease [20]. These compounds, like many other azoles, block the biosynthesis of ergosterol, which is essential for parasite survival at the level of sterol C14-demethylase (CYP51). There is, however, always interest in improving the efficacy of a given drug (decreasing the likelihood of resistance) with combination therapies. The results of this initial study support the notion that the use of posaconazole in combination with benznidazole could reduce the doses needed to obtain the same parasiticidal effect and, consequently, has the potential to diminish its side effects, the duration of therapy and/or the cost of the treatment.

High levels of *in vivo* activity of posaconazole and benznidazole, which confer almost complete protection against death to infected mice when used at doses of 20 mpk and 100 mpk, respectively, are in agreement with the activity originally reported by Filardi and Brener [29] and Molina et al. [20]. Our data are also in agreement with these reports in that treatments of infected animals with doses lower than those indicated above are unable to induce complete parasitological cures or provide protection against death, confirming the sharp dose-dependent anti-*T. cruzi* activity of these drugs. The first therapeutic scheme used in this study (7 consecutive days) allowed a fast comparative analysis of the anti-*T. cruzi* activity for each compound, making it amenable for the testing of large numbers of experimental groups, an important strategy for the evaluation of the anti-*T. cruzi* effects of drugs in combination. The data presented in Table 1, in mice infected with the Y-strain, show that a combination of half and one-fourth of the optimal doses of posaconazole and benznidazole suppressed parasitemia and reduce the mortality of the experimental animals with the same efficacy as the optimal doses of the drugs when given alone.

When a rigorous evaluation of the curative efficacy of the drugs used alone or in combination was done 30 days after treatment by immunosuppressing the treated animals and monitoring the reactivation of the Y-strain infections (Figure 1 and Table 3), it was confirmed that the combined concomitant use of sub-optimal doses of the drugs for full treatment length (20 days) or optimal doses of the drugs given sequentially for shorter treatment durations (10 days each) were able to prevent death and eradicate the parasite infection with an efficacy comparable or superior to that of the drugs when used alone at their optimal doses for the full treatment length. Interestingly, the cure rate was significantly reduced when the sequential therapy started with Ps (Ps followed by Bz). The reasons for the different cure rate detected in sequential therapy could be interpreted, at least partially, in terms of the different mechanisms of action, efficacy and time-to-kill of the two drugs. It suggests that the initial treatment with benznidazole would lead to a rapid and significant reduction of parasite biomass for subsequent action of posaconazole. The special pharmacokinetic properties of posaconazole in mammal tissue (large volume of distribution, long half - lives) would allow control of infection.

Similar conclusions were reached from the analysis of the outcomes of combined treatments against the benznidazole-resistant VL-10 strain (Figure 2 and Table 2). These results are important especially considering the limitations of each type of drug. The major limitation of benznidazole is the induction of adverse reactions that can lead to treatment discontinuation; moreover, benznidazole is genotoxic, as expected by its chemical nature and reactivity [5], while the primary disadvantage of posaconazole is the complexity and cost of manufacturing [5]. Considering the limitations of both compounds, the possibility of the reduction of doses for Chagas disease therapy is very promising, because it could lead to a reduction of both the side effects and the cost of treatment. Considering the impressive results of the sequential (benznidazole-posaconazole) treatment using shorter treatment duration (Table 3), this therapeutic scheme could be considered for evaluation in the treatment of life-threatening acute *T. cruzi* infections in humans, such as those of congenital or reactivated Chagas disease patients.

Studies using experimental models have demonstrated the efficacy of combination therapy using different pharmacological classes of compounds in Chagas disease. Benaim et al. [37] showed that the anti-arrhythmic compound amiodarone, which blocks the parasite's endogenous sterol synthesis at the level of oxidosqualene cyclase and disrupts the parasite's Ca2+ homeostasis, has a synergistic anti-*T. cruzi* activity *in vitro* and *in vivo* when used in combination with posaconazole. Araújo et al. [38] demonstrated that ketoconazole, another azole derivative that inhibits *T. cruzi* CYP51 but has no curative activity in experimental or human Chagas disease, has synergistic activity when combined with benznidazole in a murine model of acute disease. More recently, different studies have demonstrated the increased efficacy of different class of compounds when used in combination with nifurtimox [39] or benznidazole [40]. The potential of antimicrobial combination chemotherapy to improve efficacy and reduce the risk of selecting drug resistance is clear, and has been introduced in recent years for treatment of several parasitic infections, such as malaria [41], visceral leishmaniasis [42] and sleeping sickness [43].

Taken together, the results of the present study demonstrate that combinations of benznidazole and posaconazole, at sub-optimal doses or using shorter treatments, have equivalent or superior efficacy than the drugs given at their optimal doses and full treatment length in a murine model of acute Chagas disease, indicating the positive interaction of both concomitant and sequential treatments. Since both drugs are commercially available, their use in combination should be considered for evaluation in the treatment of Chagas disease patients, aiming to reduce the doses and/or the length of the treatment, hence reducing its potential toxicity (benznidazole) or cost (posaconazole).
